# Supplementary material for: Synthesis, Antimicrobial, Anti-Virulence and Anticancer Evaluation of New 5(4H)-Oxazolone-Based Sulfonamides
Source: Molecules. 2022 Jan 20;27(3):671. doi: 10.3390/molecules27030671 (PMC8838850; doi:10.3390/molecules27030671)

SUPPORTING INFORMATION FOR:

Synthesis, antimicrobial, anti-virulence and anticancer evaluation of new 5(4*H*)-Oxazolone-Based Sulfonamides.

Ahmad J. Almalki[1*], Tarek S. Ibrahim[1,2], Ehab S. Taher[3], Mamdouh F. A. Mohamed[4], Mahmoud Youns[5], Wael A.H. Hegazy[6] and Amany M. M. Al-Mahmoudy[2*].

[1] Department of Pharmaceutical Chemistry, Faculty of Pharmacy, King Abdulaziz University, Jeddah, 21589, Saudi Arabia. **ajalmalki@kau.edu.sa** (A.J.A); tmabrahem@kau.edu.sa (T.S.I)

[2] Department of Pharmaceutical Organic Chemistry, Faculty of Pharmacy, Zagazig University, Zagazig, 44519, Egypt. tmabrahem@kau.edu.sa (T.S.I); amanysinger77@gmail.com (A.M.M.A.)

[3] Department of Pharmaceutical Organic Chemistry, Faculty of Pharmacy, Al-Azhar University, Assiut, 71524, Egypt. ehabtaher@azhar.edu.eg (E. S. T)

[4] Department of Pharmaceutical Chemistry, Faculty of Pharmacy, Sohag University, 82524 Sohag, Egypt. mamdouh.fawzi@pharm.sohag.edu.eg (M.F.A.M)

[5] Department of Biochemistry and Molecular Biology, Faculty of Pharmacy, Helwan University, Cairo, Egypt. Mahmoud_younis@pharm.helwan.edu (M.Y.).

[6] Department of Microbiology and Immunology, Faculty of Pharmacy, Zagazig University- Zagazig- Egypt, waelmhegazy@daad-alumni.de (W.A.H.H).

* Correspondence: **ajalmalki@kau.edu.sa** (A.J.A); amanysinger77@gmail.com (A.M.M.A.)

CONTENTS **Page**

^{1}H and ^{13}C NMR Spectra for Compounds 9a-k **2**

^{1}H NMR for compound 9a

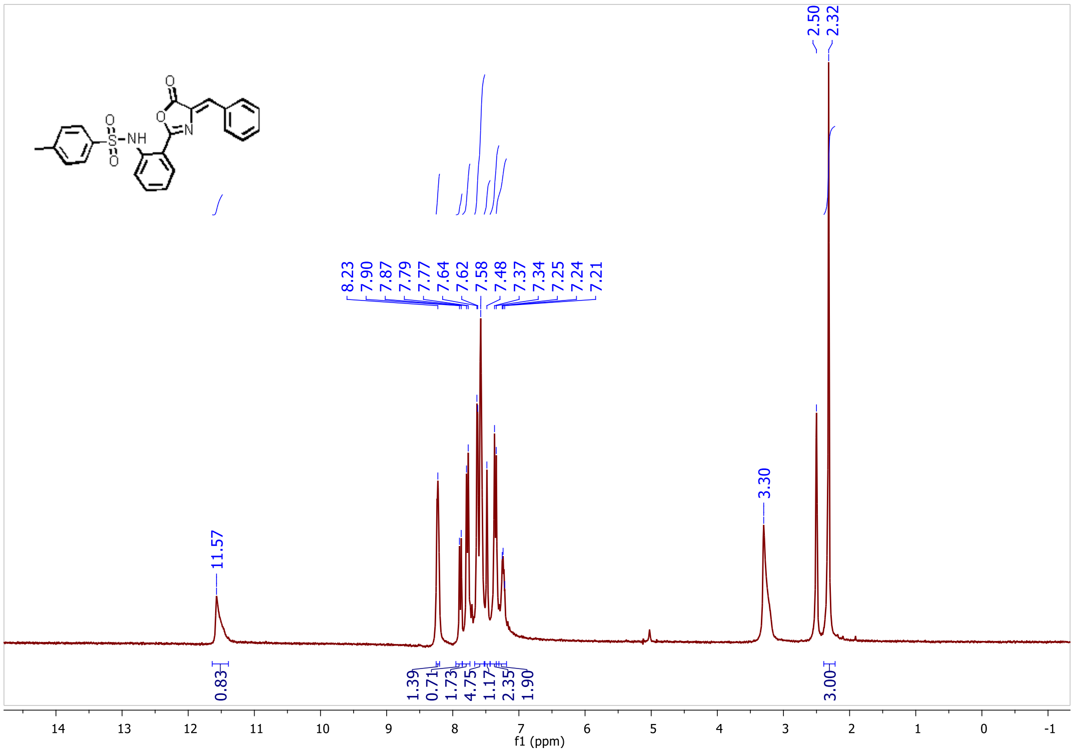

^{13}C NMR for compound 9a

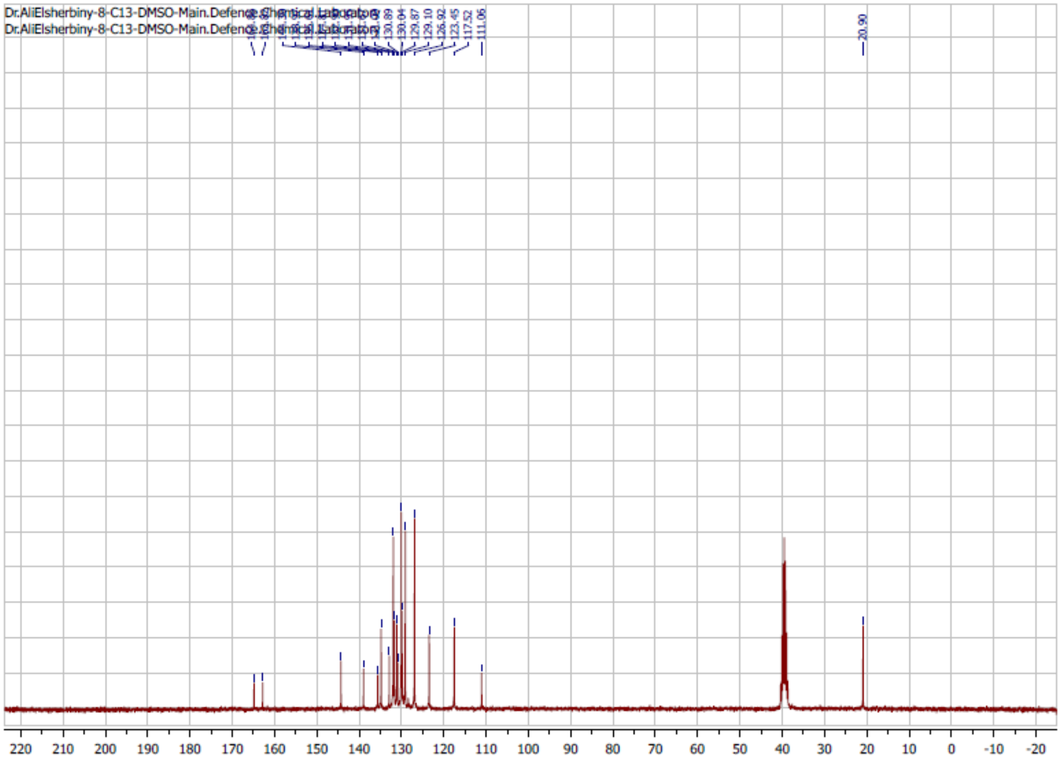

^{1}H NMR for compound 9b

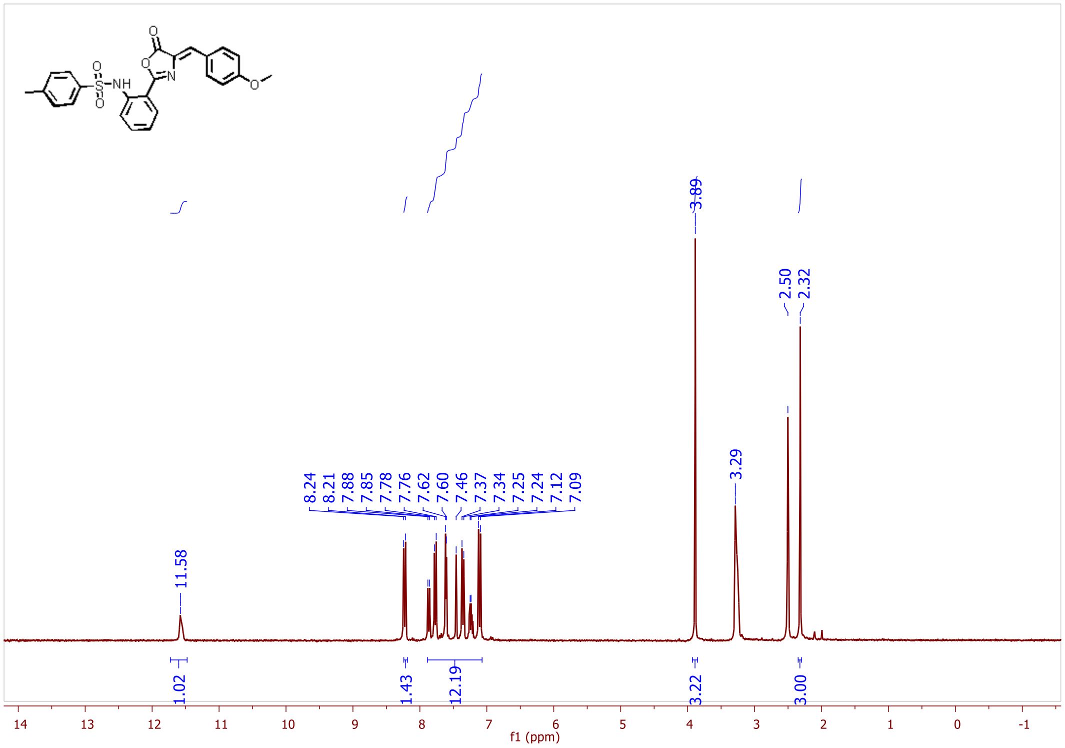

^{13}C NMR for compound 9b

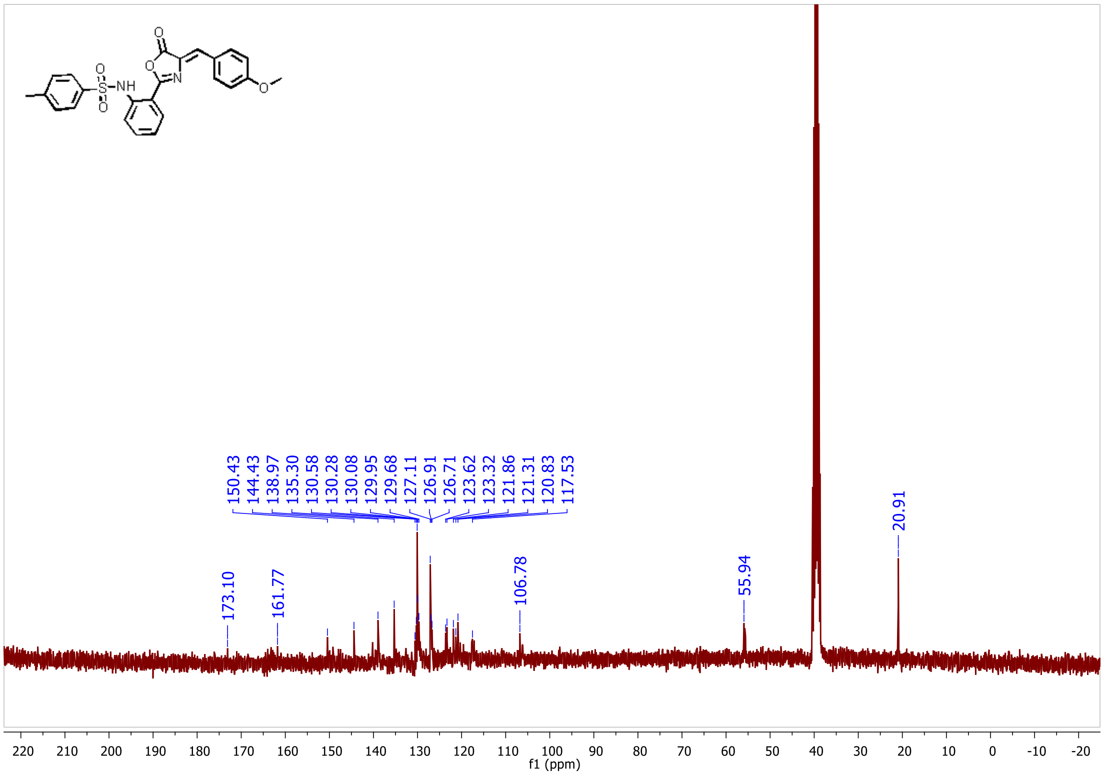

^{1}H NMR for compound 9c

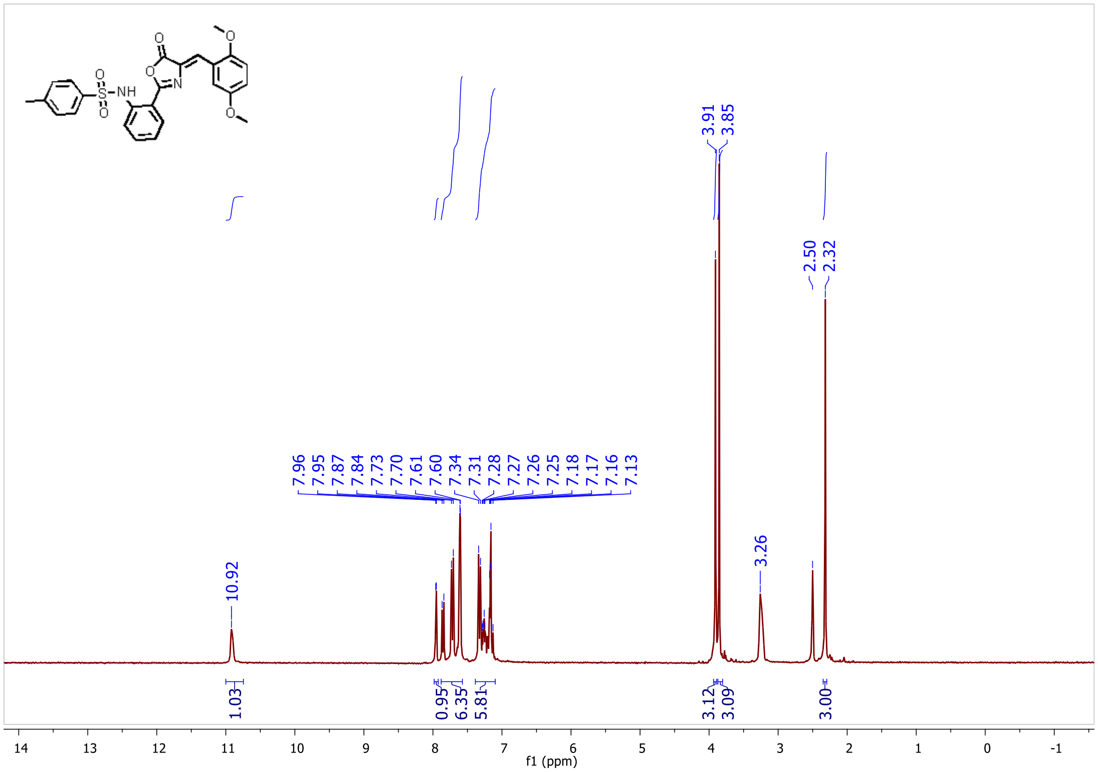

^{13}C NMR for compound 9c

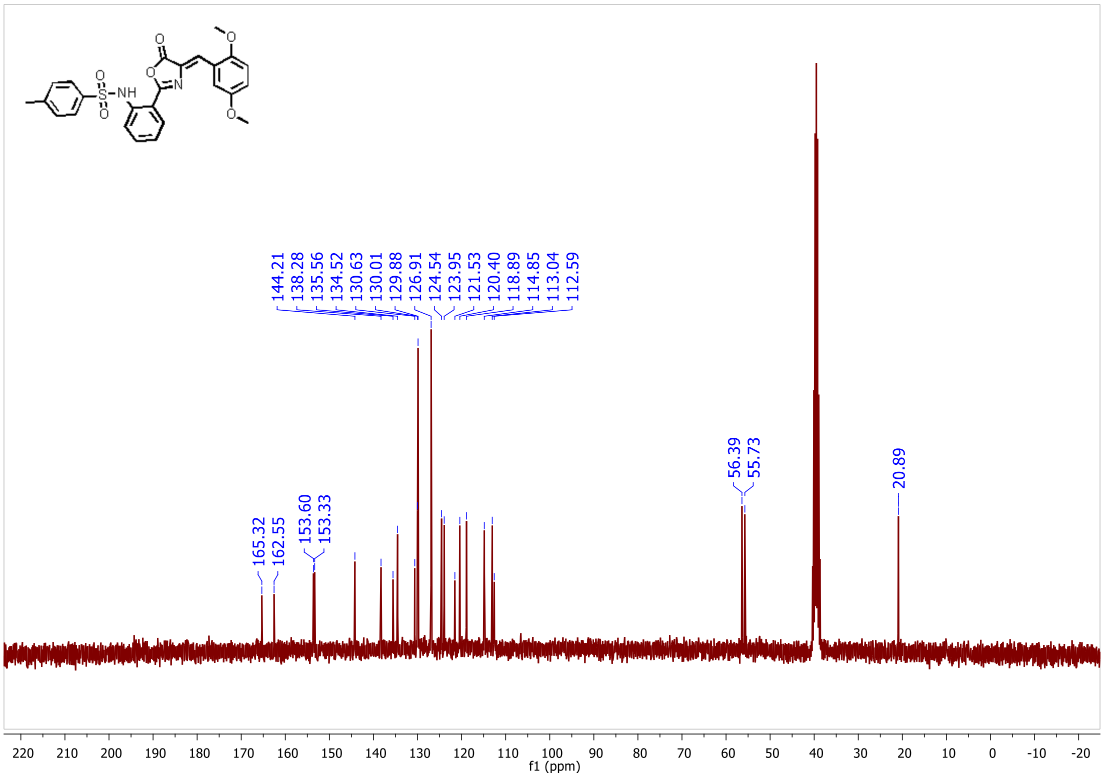

^{1}H NMR for compound 9d

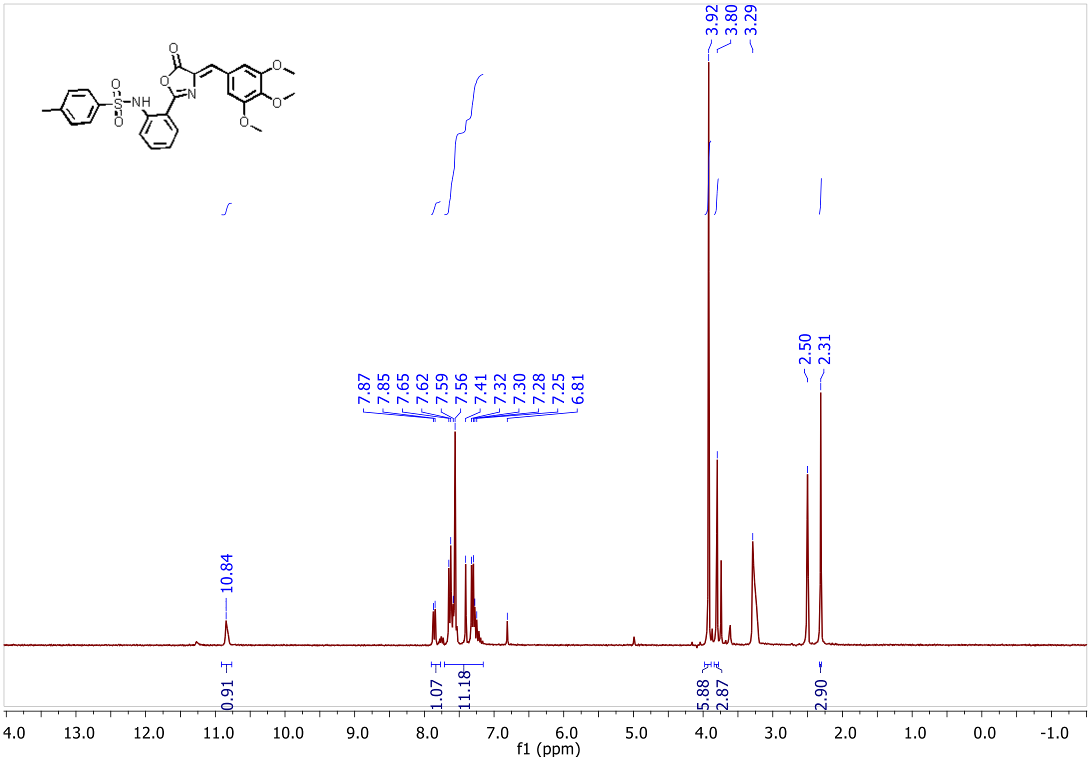

^{13}C NMR for compound 9d

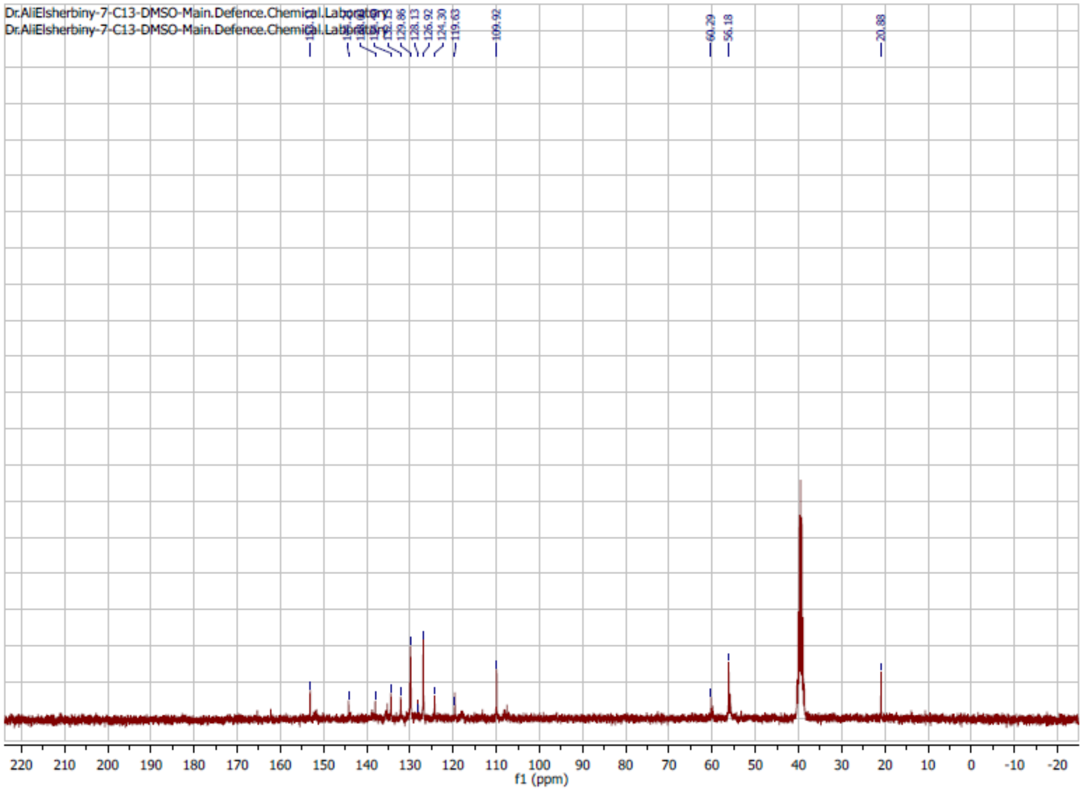

^{1}H NMR for compound 9e

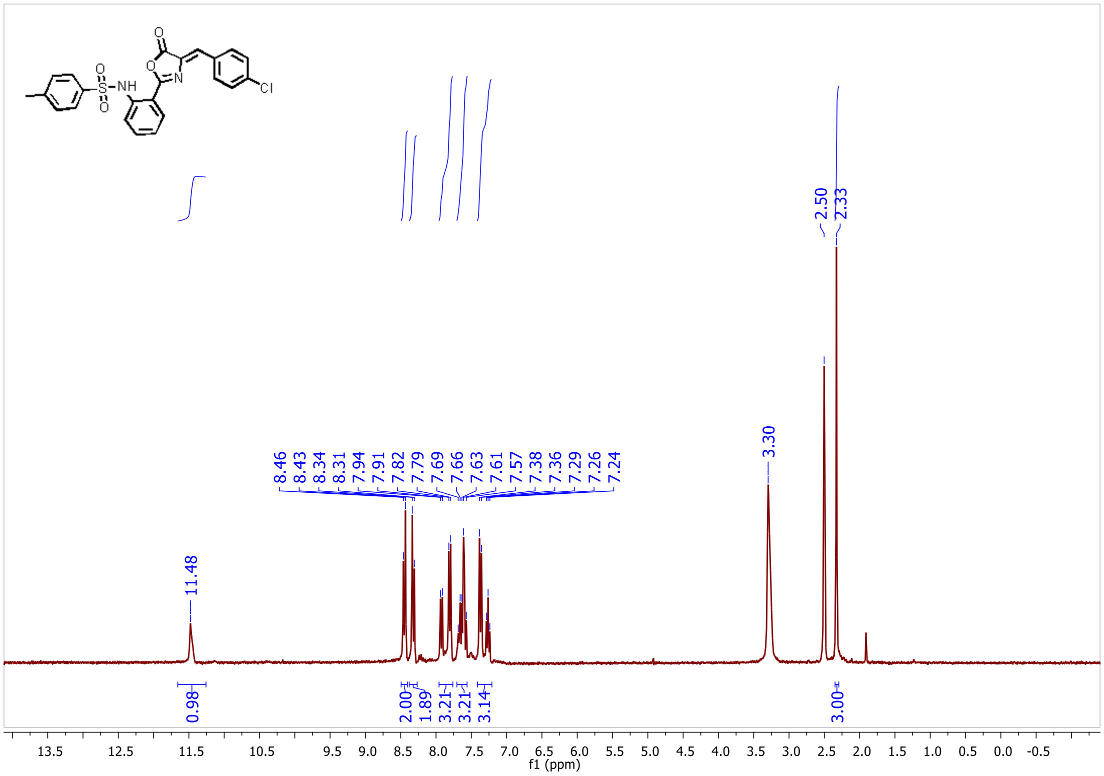

^{13}C NMR for compound 9e

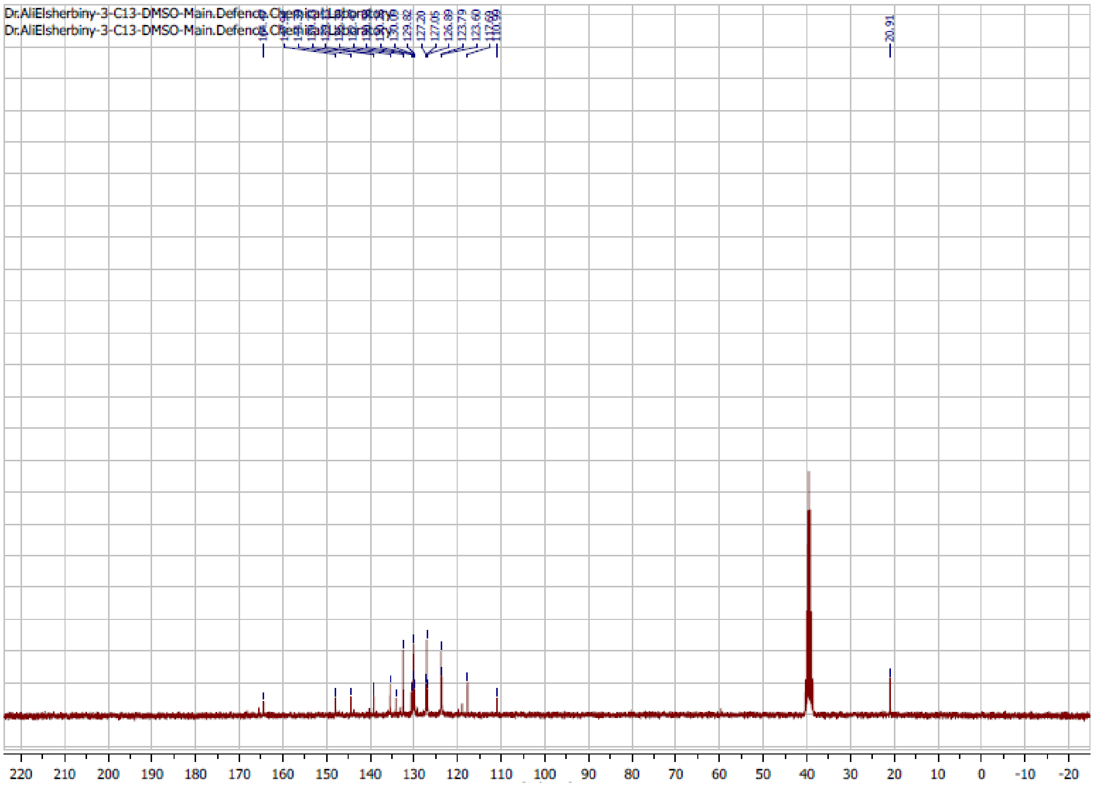

^{1}H NMR for compound 9f

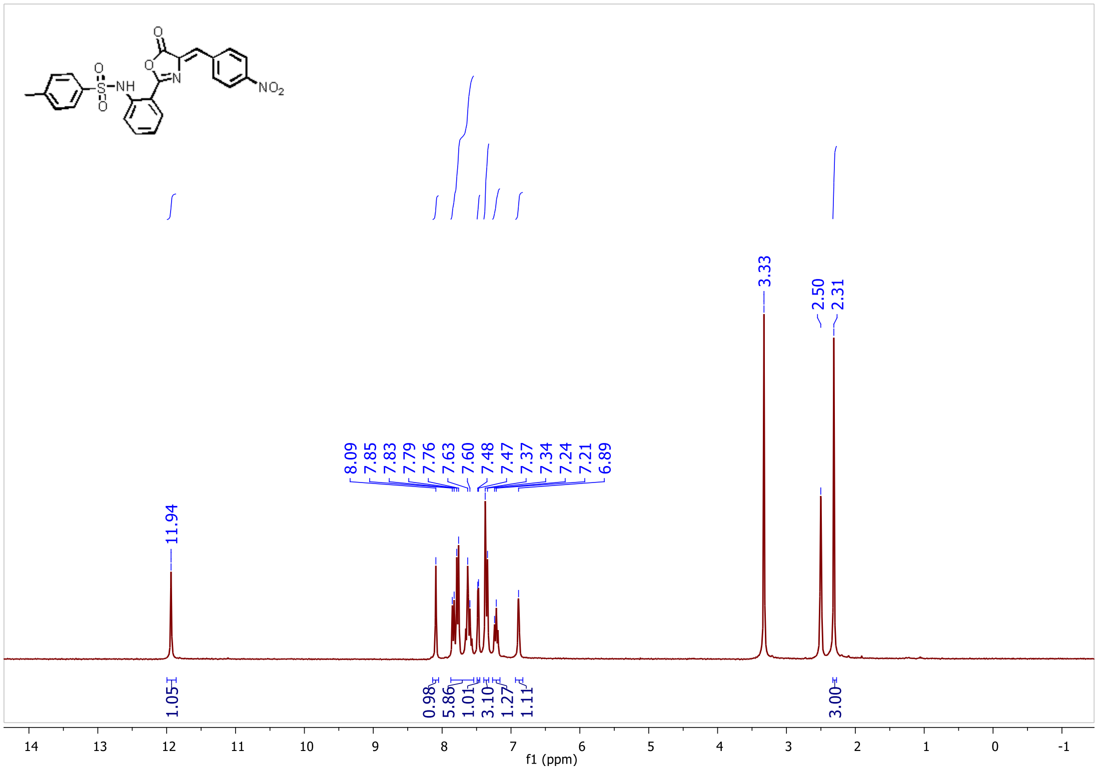

^{13}C NMR for compound 9f

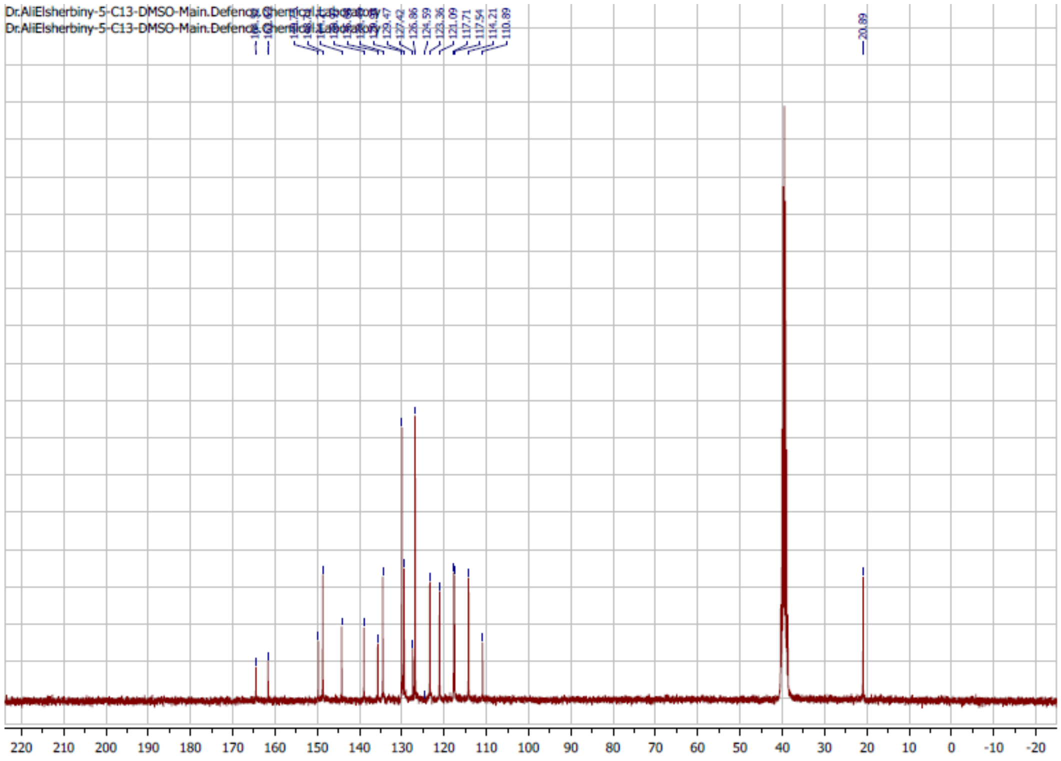

^{1}H NMR for compound 9g

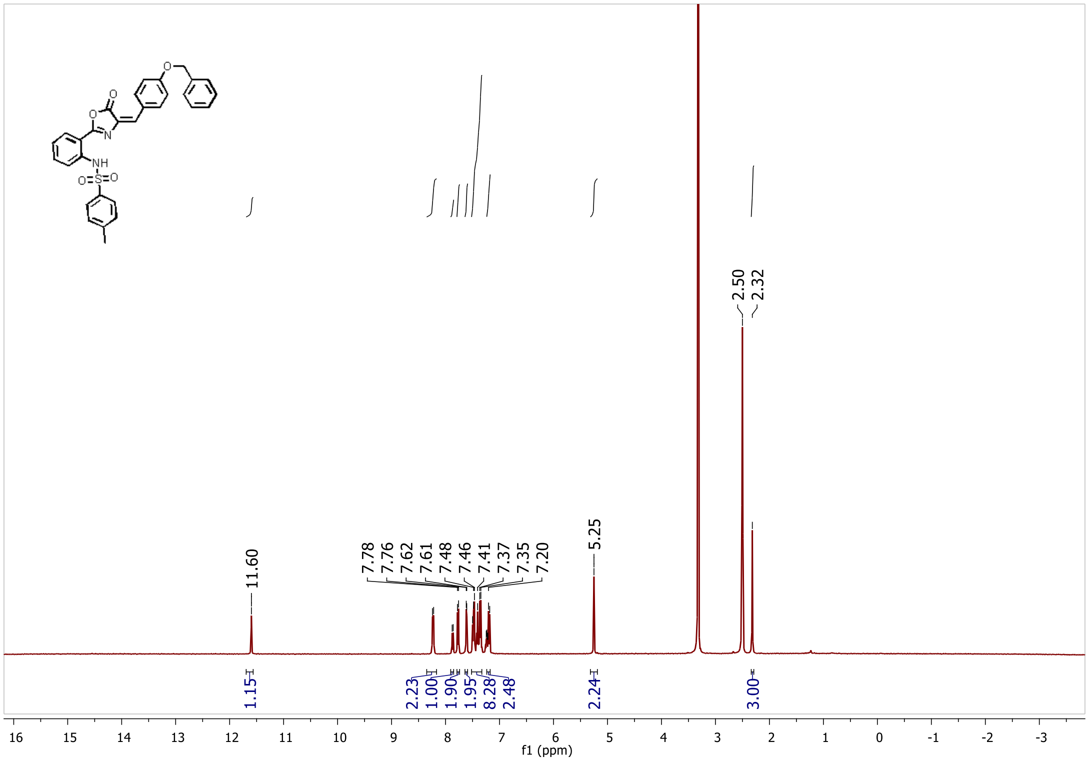

^{13}C NMR for compound 9g

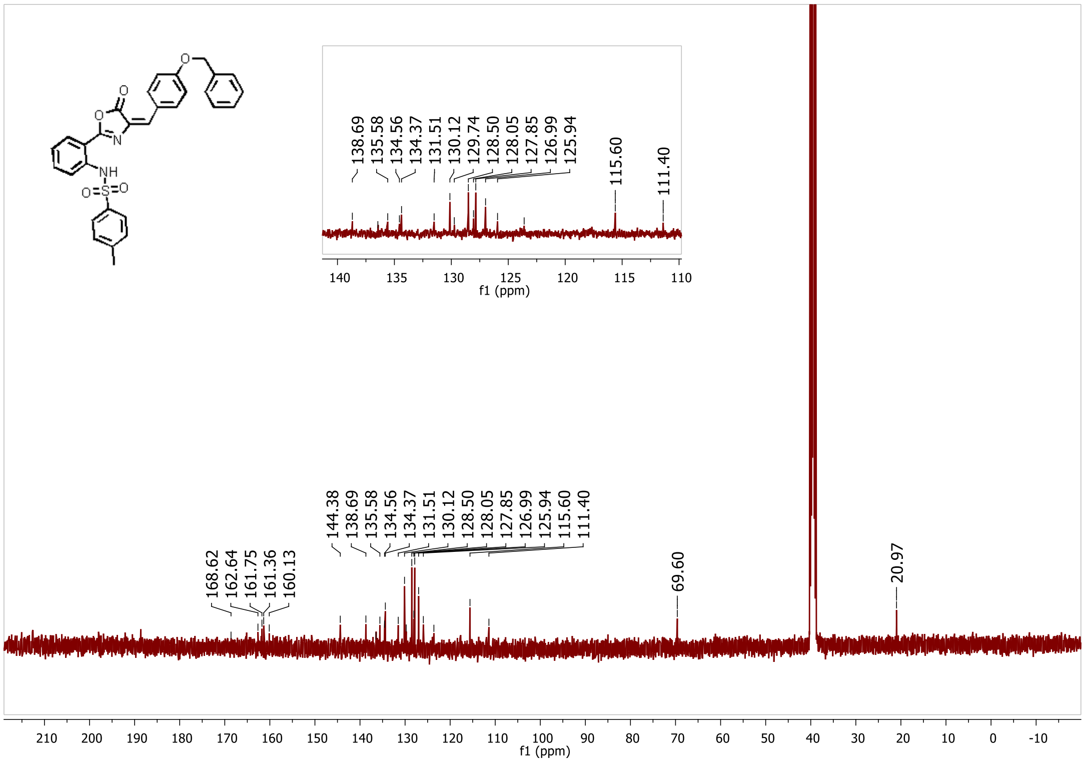

^{1}H NMR for compound 9h

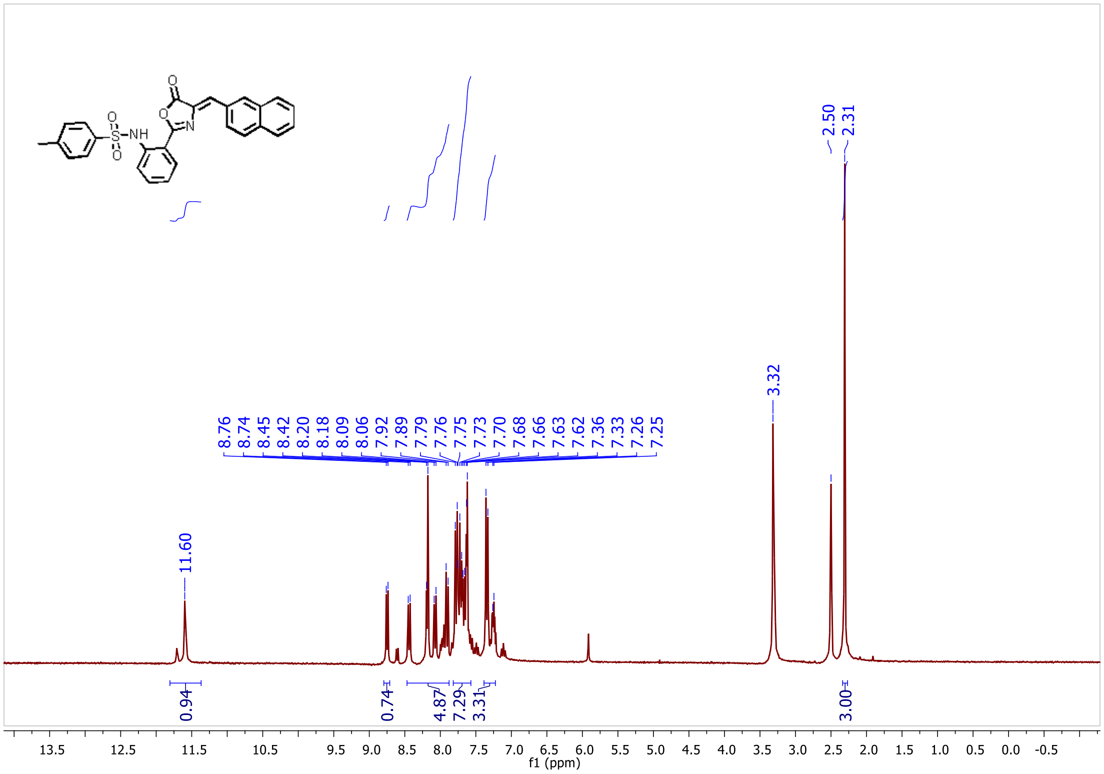

^{13}C NMR for compound 9h

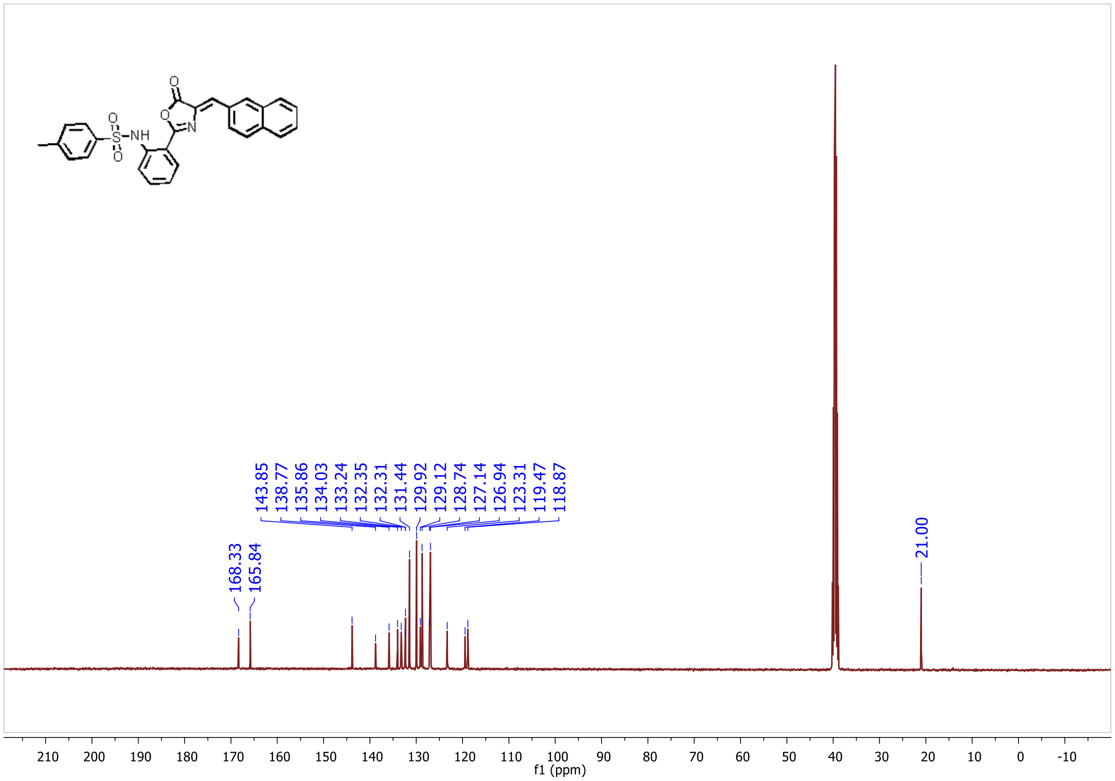

^{1}H NMR for compound 9i

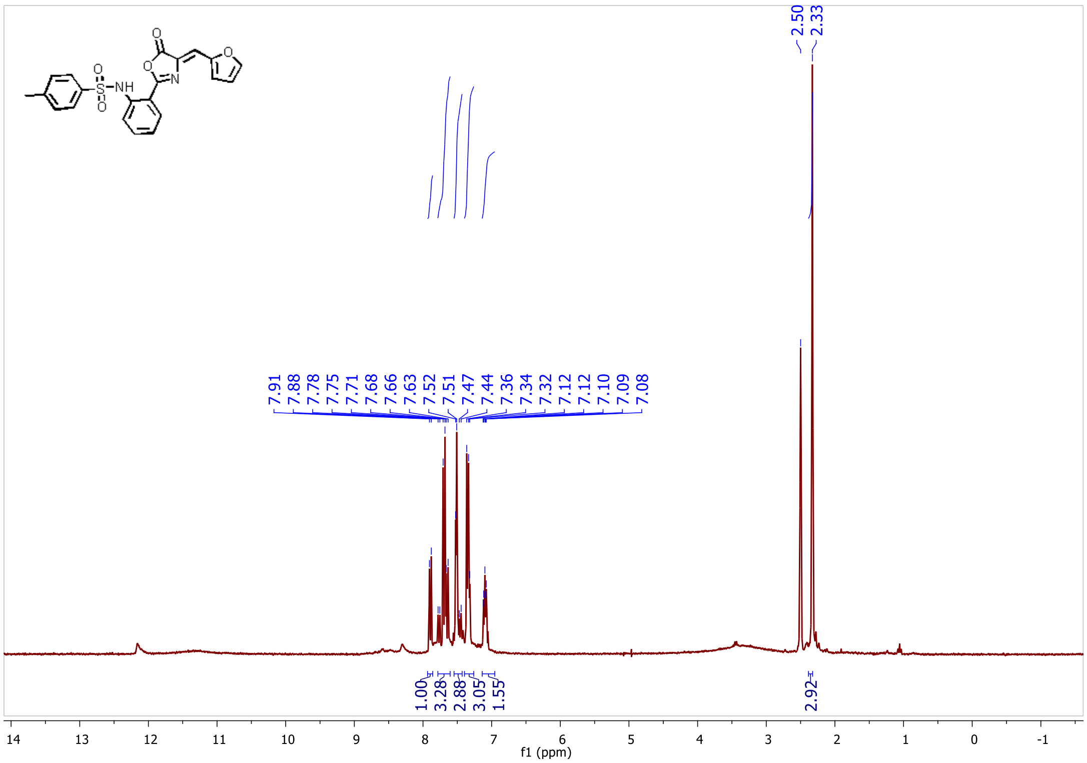

^{13}C NMR for compound 9i

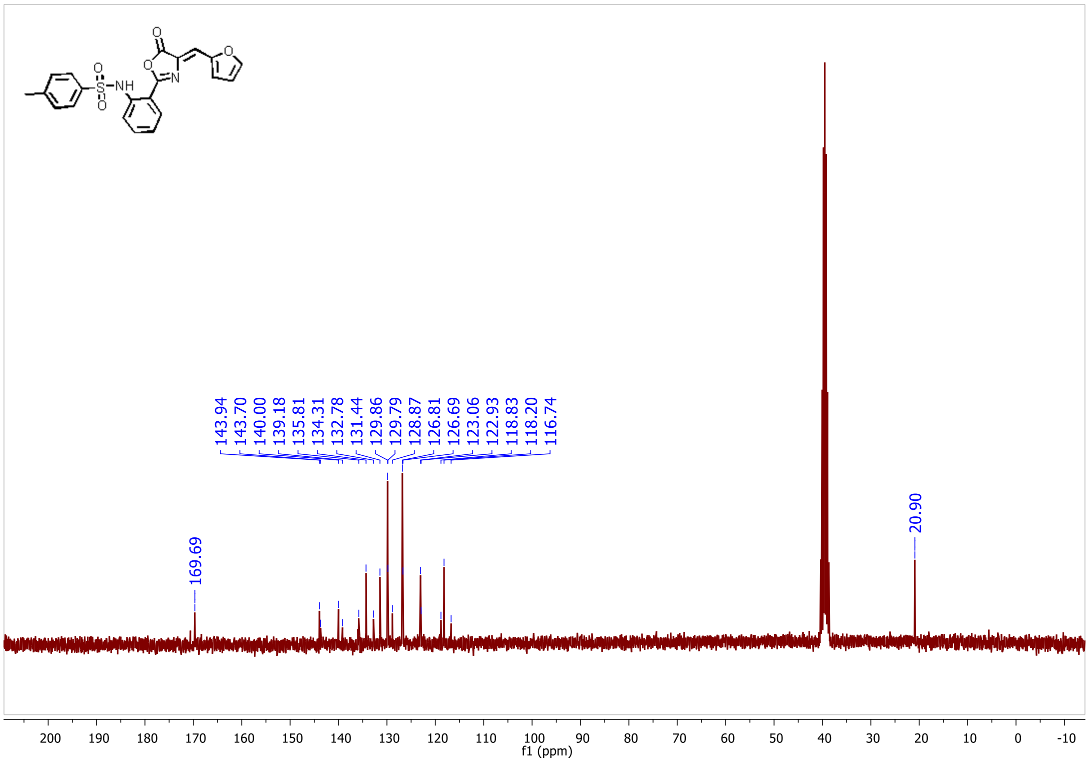

^{1}H NMR for compound 9j

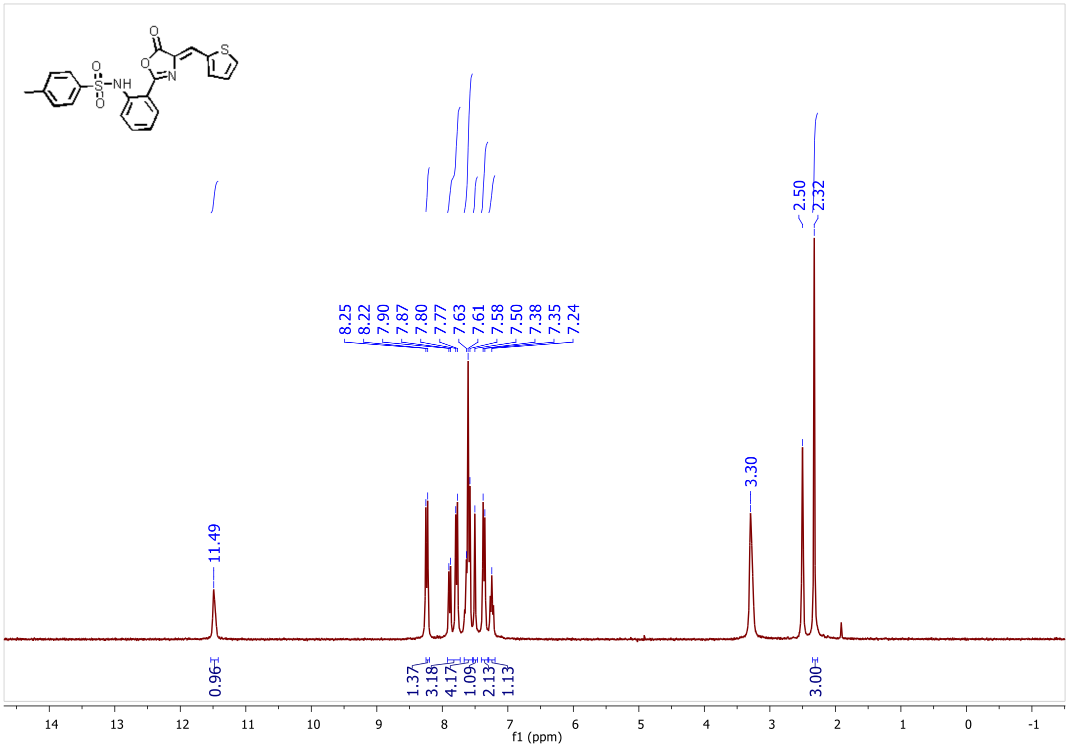

^{13}C NMR for compound 9j

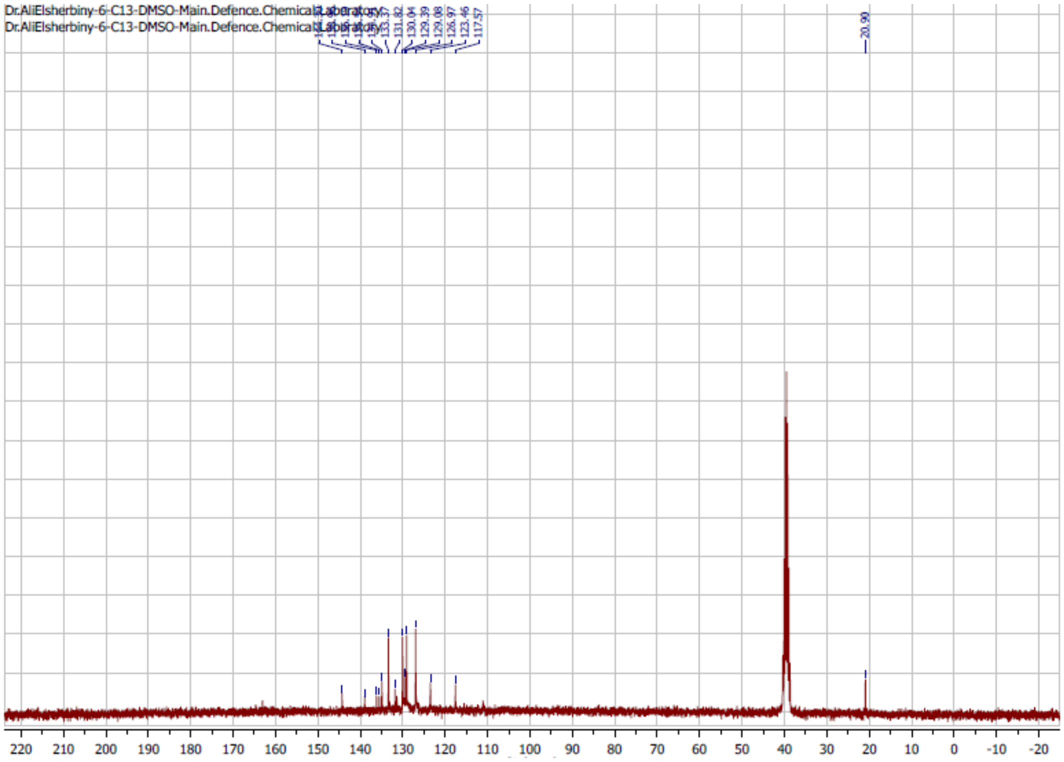

^{1}H NMR for compound 9k

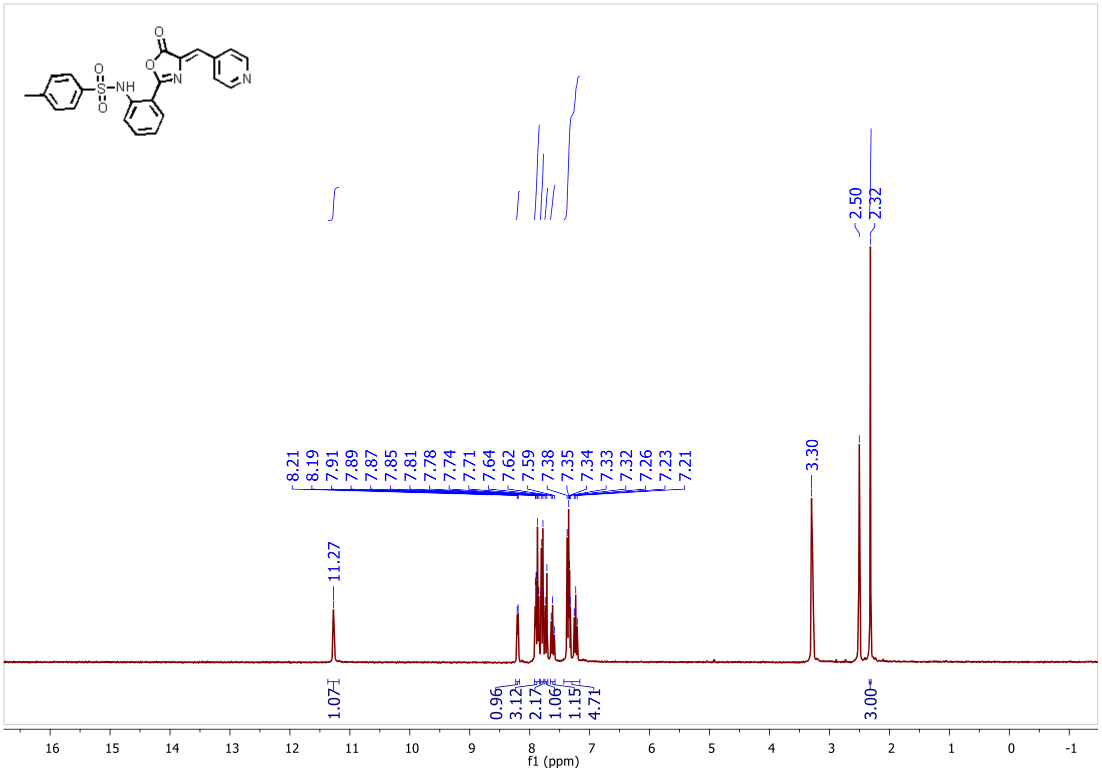

^{13}C NMR for compound 9k

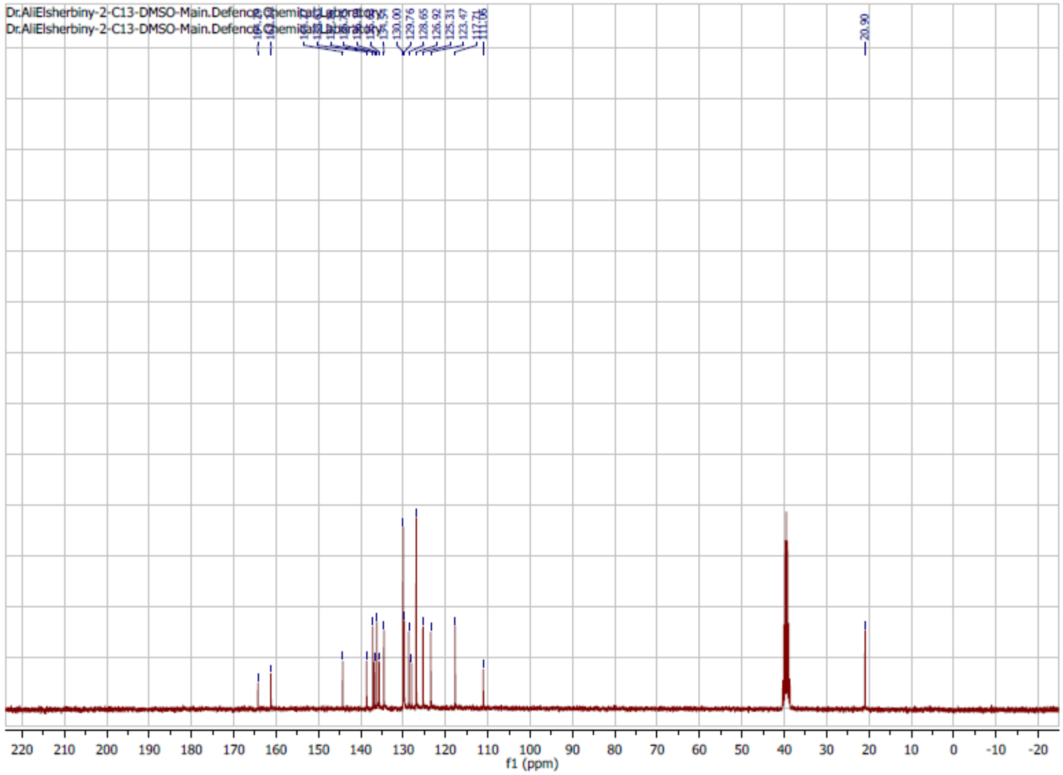

Supplement: Supplementary file 1 [file molecules-27-00671-s001.zip › molecules-1559916-supplementary.pdf]
